# MRI distortion correction is associated with improved local control in stereotactic radiotherapy for brain metastases

**DOI:** 10.1038/s41598-025-93255-3

**Published:** 2025-03-17

**Authors:** Daniel Höfler, Johanna Grigo, Hadi Siavosch, Marc Saake, Manuel Alexander Schmidt, Thomas Weissmann, Philipp Schubert, Raphaela Voigt, Sebastian Lettmaier, Sabine Semrau, Arnd Dörfler, Michael Uder, Christoph Bert, Rainer Fietkau, Florian Putz

**Affiliations:** 1 Department of Radiation Oncology, Universitätsklinikum Erlangen, Friedrich-Alexander-Universität Erlangen-Nürnberg, Universitätsstraße 27, 91054 Erlangen, Germany; 2 Institute of Radiology, Universitätsklinikum Erlangen, Friedrich-Alexander-Universität Erlangen-Nürnberg, Ulmenweg 18, Erlangen, Germany; 3 Department of Neuroradiology, Universitätsklinikum Erlangen, Friedrich-Alexander-Universität Erlangen-Nürnberg, Schwabachanlage 6, Erlangen, Germany; 4 Comprehensive Cancer Center Erlangen-EMN (CCC ER-EMN), Erlangen, Germany; 5 Bavarian Cancer Research Center (BZKF), Munich, Germany

**Keywords:** Radiotherapy, Cancer imaging, CNS cancer

## Abstract

Distortions in brain MRI caused by gradient nonlinearities may reach several millimeters, thus distortion correction is strongly recommended for radiotherapy treatment planning. However, the significance of MRI distortion correction on actual clinical outcomes has not been described yet. Therefore, we investigated the impact of planning MRI distortion correction on subsequent local control in a historic series of 419 brain metastases in 189 patients treated with stereotactic radiotherapy between 01/2003 and 04/2015. Local control was evaluated using a volumetric extension of the RANO-BM criteria. The predictive significance of distortion correction was assessed using competing risk analysis. In this cohort, 2D distortion-corrected MRIs had been used for treatment planning in 52.5% (220/419) of lesions, while uncorrected MRIs had been employed in 47.5% (199/419) of metastases. 2D distortion correction was associated with improved local control (Cumulative incidence of local progression at 12 months: 14.3% vs. 21.2% and at 24 months: 18.7% vs. 28.6%, p = 0.038). In multivariate analysis, adjusting for histology, baseline tumor volume, interval between MRI and treatment delivery, year of planning MRI, biologically effective dose and adjuvant Whole-brain radiotherapy, use of distortion correction remained significantly associated with improved local control (HR 0.55, p = 0.020). This is the first study to clinically evaluate the impact of MRI gradient nonlinearity distortion correction on local control in stereotactic radiotherapy for brain metastases. In this historic series, we found significantly higher local control when using 2D corrected vs. uncorrected MRI studies for treatment planning. These results stress the importance of assuring that MR images used for radiotherapy treatment planning are properly distortion-corrected.

## Introduction

Brain metastases constitute the most common malignancy of the brain and occur in up to 40% of patients with cancer^1^. Stereotactic radiotherapy is one of the most important treatment modalities in brain metastases due to its high efficacy, low patient burden and favorable toxicity-profile^2^. While brain metastases used to carry a dismal prognosis in the past, today an ever-growing number of patients achieve long-term survival, shifting the main focus of stereotactic radiotherapy from short- or mid-term palliation to definitive long-term control^3,4^. In addition, radiation oncologists are increasingly reserved about recommending adjuvant whole-brain radiotherapy because of potential side effects on cognition and quality of life^5,6^. However, omitting adjuvant whole-brain radiation not only increases distant brain failure but also reduces local control^6–8^.

It is therefore ever more important to improve and optimize the efficacy of brain stereotactic radiotherapy. Stereotactic radiotherapy relies on high geometric accuracy to deliver an ablative dose to small targets while sparing surrounding normal brain tissue. Highly precise treatment planning and delivery depends on an accurate and up to date representation of the tumor geometry on magnetic resonance imaging (MRI) as errors introduced in pretreatment imaging can hardly be corrected in subsequent steps^9,10^. Previous studies have conclusively demonstrated that pretreatment imaging significantly influences treatment outcomes. Specifically, longer intervals between pretreatment MRI and stereotactic radiosurgery, leading to inaccuracies in gross target volume (GTV), have been linked to decreased control of brain metastasis^11–14^.

While images from computed tomography (CT) can be considered geometrically accurate, multiple types of image distortions occur in MRI. In MRI, spatial encoding is achieved by linearly varying gradients of magnetic field strength in the X, Y and Z-direction that are generated by dedicated gradient coils^15–20^. However, because of gradient coil design, gradient nonlinearities are present especially at the periphery of the scanner^19,21^. These nonlinearities lead to distortions in reconstructed images that increase with radial distance from the isocenter and may reach several millimeters at the periphery of the brain^18,21–24^. Vendor-specific 2D and 3D distortion corrections are available at most modern scanners, with 3D distortion correction also being able to correct for through-plane distortions^25^. In recent consensus recommendations, 3D distortion correction of MR images was therefore considered a mandatory requirement for MRI-based radiotherapy treatment planning^9^. However, the issue of assuring proper distortion correction in MR images is, in our experience, still not widely recognized in the radiation oncology community. In the context of the large body of literature that has measured distortions in MR images^15–17,19,21^, Seibert et al. recently observed, in a simulation study, that uncorrected MR images would have led to geographic miss in 8 out of 28 metastases^26^. Yet, the actual clinical relevance of distortion correction and real impact on local control in stereotactic radiotherapy of brain metastases remain undemonstrated. In the present study, we compare local control achieved with corrected and uncorrected planning MRI datasets to evaluate the clinical significance of MRI distortion correction in brain stereotactic radiotherapy.

## Methods

### Ethics statement

The requirement for ethical approval of this retrospective study was waived by the licensing authority (federal legislation GDNG Art. 6 (1); state legislation BayKrG Art. 27 (4)) and in accordance with institutional guidelines. This retrospective study followed the 1964 Declaration of Helsinki and its later amendments. All patients provided informed consent for treatment.

### Patient population

To obtain the cohort for this analysis, we identified all patients who received stereotactic radiotherapy (SRT) for intracranial metastases at our institution between January 2003 and April 2015, before an optimized brain planning MRI protocol was implemented. From this group of 566 patients, we selected radiation treatment plans based on the following inclusion criteria: (1) stereotactic radiotherapy for intraparenchymal brain metastases from a solid cancer, (2) no prior SRT and no prior resection of the target metastasis, (3) Contrast-enhanced T1-weighted magnetization-prepared rapid gradient-echo images (T1-MPRAGE) sequence with ≤ 1 mm slice thickness included in planning MRI and conducted at least once during aftercare. A total of 419 brain metastases in 189 patients fulfilled these criteria and were selected for further analysis. From these 419 brain metastases, 2D distortion-corrected MRIs had been used for treatment planning in 52.5% (220/419) of lesions, while uncorrected MRIs had been employed in the remaining 47.5% (199/419) metastases. No instance of 3D distortion correction was identified in the present series. Table 1 lists further characteristics of both subsets and the total cohort.

**Table 1:** Characteristics of treated brain metastases.

Metastasis characteristic	Total cohort (N = 419)	2D correction (N = 220)	No correction (N = 199)	*p* value
Interval between MRI and first treatment delivery, days				
Median (IQR)	9 (6–18)	9 (6–20)	9 (6–18)	0.504^#^
Primary cancer histology, n (%)				0.014*
Melanoma^R^	178 (42.5%)	80 (36.4%)	98 (49.2%)	
Lung	93 (22.2%)	58 (26.4%)	35 (17.6%)	
Breast	52 (12.4%)	23 (10.5%)	29 (14.6%)	
Renal^R^	44 (10.5%)	30 (13.6%)	14 (7.0%)	
Gastrointestinal	23 (5.5%)	16 (7.3%)	7 (3.5%)	
Bladder / Urinary tract	6 (1.4%)	2 (0.9%)	4 (2.0%)	
Sarcoma^R^	7 (1.7%)	3 (1.4%)	4 (2.0%)	
Gynecologic	4 (1.0%)	1 (0.5%)	3 (1.5%)	
Other	12 (2.9%)	7 (3.2%)	5 (2.5%)	
Primary cancer histology, n (%)				0.202*
Radioresistant histology	228 (54.4%)	113 (51.4%)	115 (57.8%)	
Non-radioresistant histology	191 (45.6%)	107 (48.6%)	84 (42.2%)	
Pretreatment metastasis volume, cm^3^				0.297^#^
Median (IQR)	0.29 (0.08–1.25)	0.22 (0.06–1.12)	0.36 (0.11–1.48)	
Adjuvant WBRT, n (%)				0.197*
No	246 (58.7%)	136 (61.8%)	110 (55.3%)	
Yes	173 (41.3%)	84 (38.2%)	89 (44.7%)	
Type of stereotactic radiotherapy, n (%)				0.032*
Single session radiosurgery (SRS)	215 (51.3%)	124 (56.4%)	91 (45.7%)	
Fractionated stereotactic radiotherapy (FSRT)	204 (48.7%)	96 (43.6%)	108 (54.3%)	
SRS Single dose, Gy				0.987^#^
Median (IQR)	18.0 (18.0–20.0)	18.0 (18.0–20.0)	18.0 (18.0–20.0)	
FSRT: Dose per fraction, Gy				< 0.001^#^
Median (IQR)	4.0 (3.0–4.0)	4.0 (3.0–4.0)	4.0 (4.0–6.0)	
FSRT: Total dose, Gy				0.051^#^
Median (IQR)	30.0 (20.0–40.0)	35.0 (21.0–40.0)	30.0 (20.0–35.0)	
Total BED_12-LQC_, Gy				0.100^#^
Median (IQR)	52.4 (41.0–72.6)	52.4 (41.0–69.2)	52.8 (41.0–72.6)	
Min. distance from brain surface, mm				0.037^#^
Median (IQR)	5.7 (1.4–14.6)	6.2 (2.0–16.1)	4.8 (1.1–12.8)	
Metastasis location, n (%)				0.026*
Central	312 (74.5%)	173 (78.6%)	139 (69.8%)	
Peripheral	107 (25.5%)	47 (21.4%)	60 (30.2%)	
Field strength, n (%)				< 0.001*
1.5 T	379 (90.5%)	180 (81.8%)	199 (100%)	
3.0 T	40 (9.5%)	40 (18.2%)	0 (0%)	

Total BED_12-LQC_ was calculated including WBRT dose, if upfront WBRT was delivered prior to stereotactic radiotherapy.

*IQR* interquartile range, *WBRT* Whole-brain radiotherapy, *BED*_*12-LQC*_ Biologically effective dose for an alpha/beta ratio of 12, linear-quadratic-cubic model, *Min* Minimum.

Rclassified as radioresistant histology #T-Test *Fisher’s exact test.

### Radiation therapy

Patients received single-session radiosurgery (SRS) or fractionated stereotactic radiotherapy (FSRT) with a linear-accelerator based Novalis® or Novalis-Tx® system (BrainLAB, Feldkirchen, Germany). Patients were immobilized in an individually manufactured thermoplastic head mask attached to a stereotactic base frame (BrainLAB, Feldkirchen, Germany). Treatment planning was performed using Iplan (BrainLAB, Feldkirchen, Germany)^27,28^.

Patients received a dedicated planning CT, which was rigidly coregistered with the diagnostic baseline MRI using the Iplan software based on a mutual information-based algorithm. The GTV was delineated in the contrast-enhanced T1-MPRAGE sequence of the baseline MRI study. PTV was defined as the GTV with a margin of 1 mm. As clinical standard, doses were prescribed to the PTV-encompassing 80%-isodose normalized to the maximum dose (Dmax = 100%). The dose calculation grid size was 2 mm or finer according to ICRU 91. Stereotactic radiotherapy was delivered using stereotactic arcs. During treatment, daily stereoscopic X-ray imaging (ExacTrac®) was used for setup verification and repositioning. For SRS, stereoscopic X-ray imaging was repeated after every couch rotation.

Of all metastases, 41.3% (173/419) had undergone Whole-brain radiotherapy (WBRT) before stereotactic radiotherapy (SRT) while 58.7% (246/419) received SRT alone. Median WBRT fraction dose was 3 Gy (interquartile range [IQR], 2–3 Gy) and median total WBRT dose was 40 Gy (IQR, 30–40 Gy). The most prescribed fractionation schemes were 10 × 3 Gy (87/173, 50.3%) and 20 × 2 Gy (57/173, 32.9%). In the case of upfront WBRT, WBRT was considered integral part of the treatment and the start date of WBRT was determined to be the start of radiotherapy for the respective brain metastases. In addition, WBRT dose was included in the calculation of the biologically effective dose.

Among all metastases, 51.3% (215/419) were treated with SRS while 48.7% (204/419) were treated with FSRT. As established by Wiggenraad et al., biologically effective dose (BED) was calculated based on an α/β ratio of 12 according to the LQC model (BED_12-LQC_)^29,30^:

$$BED_{{12{-}LQC}} = nd \left[ {1 + \frac{d}{{\left( {\frac{\alpha }{\beta }} \right)}} - \frac{{d^{2} }}{{\left( {\frac{\alpha }{\gamma }} \right)}}} \right]$$

With *n* being the number of fractions and *d* being the dose per fraction, α/β was assumed to be 12 Gy and α/γ 648 Gy^2^^29,30^.

In case of upfront WBRT, BED_12-LQC_ were separately calculated for WBRT and SRT and added together to form the total BED_12-LQC_ used for further calculations.

### Imaging and follow-up

Images were collected on different Siemens 1.5 and 3 Tesla MRI scanners by two radiology departments within our institution. MRI protocols were defined locally in every scanner software. Therefore, distortion correction was not standardized across scanners and departments. Patients were assigned to departments and scanners depending on the availability of appointments. No restrictions regarding magnetic field strength or MR scanner type were applied during the time of the study. All analyzed images consisted of 160 or 192 contiguous, sagittal or transversal planes of 3-dimensional T1-MPRAGE with slice thickness ≤ 1 mm after intravenous application of standard-dose gadolinium-based contrast agent. In Siemens MRI scanners, gradient nonlinearity distortion correction is enabled as a post-processing step^22,31^ and the type of distortion correction is written into the DICOM header (DICOM tag 0008,0008) of exported images. For this study, we reviewed all DICOM file headers of MRI studies used for radiotherapy treatment planning to classify them into uncorrected (“ND”) and 2D distortion-corrected (“2D”).

### Volumetric extension of the RANO-BM criteria for the assessment of progression

In total, 3145 MRI studies were used for volumetric analysis (median of 6, IQR 4–9 per patient). Semiautomatic segmentation was performed using the open-source software 3D Slicer (version 4.5.0) as described previously^32^. Progression was assessed using a volumetric extension of RANO-BM criteria as described before^32,33^. Local progression was defined as ≥ 72.8% increase in volume in the present study relative to nadir/baseline, which corresponds to a ≥ 20% increase in diameter of a perfect sphere (i.e. the unidimensional RANO-BM criteria for progression). An additional absolute increase in volume of at least 0.2 cm^3^ was required for the definition of progression. This corresponds to the absolute volume increase of a 5 mm sphere growing additional 3 mm in diameter and is derived from the unidimensional RANO-BM criteria for lesions < 10 mm in diameter. Lesions meeting the volumetric criteria for progression but subsequently demonstrating spontaneous regression on follow-up imaging were classified as pseudo-progression rather than progression. Spontaneous regression was defined as a return to baseline/nadir volume or achieving a volumetric partial response, as outlined in the RANO-BM guidelines (i.e., a reduction in volume of ≥ 65%) without any additional local or systemic treatments that could account for tumor shrinkage. Enlarging lesions requiring surgical resection were classified based on histology^32,33^. Change in distant lesions, corticosteroid use or clinical status or other non-imaging criteria were not considered in the definition of progression in the present study.

### Statistical analysis

Local progression was assessed using the cumulative incidence method to account for the competing risk of death^34^. Time-to-event metrics were calculated from the first day of radiotherapy until progression, death or loss of follow-up. Local control was compared between the 2D and uncorrected subgroup using Gray’s test^35^. A proportional subdistribution hazards regression model^36^ was used to account for other known prognosticators^37^ of local control in multivariate analysis. As common in brain metastases, melanoma, renal cell cancer and sarcoma were considered radioresistant histologies^38^. Overall survival was calculated at patient level from the date of the first stereotactic radiotherapy until death or loss to follow-up and evaluated according to the Kaplan–Meier method^39^. Differences in overall survival between patients with 2D-corrected and uncorrected planning MRI at first stereotactic radiotherapy were assessed by the log rank test^40,41^. The location of metastases in reference to the brain surface was automatically calculated. In brief, the brain was auto-segmented using the HD-BET deep learning pipeline^42^, subsequently the brain and brain metastasis label map segmentations were converted into meshes using the 3DSlicer^43^ segmentation module and finally the minimum distance between the brain and tumor surface was calculated using VTK DistancePolyDataFilter^44^. Metastases with a minimum distance of 1.5 cm or less from the brain surface were considered peripheral and all other tumors were considered centrally located.

P-values < 0.05 were considered statistically significant. Statistical analyses were performed using IBM SPSS 21 and R statistical software (3.5.2) using the R package cmprsk.

## Results

**Fig. 1:**
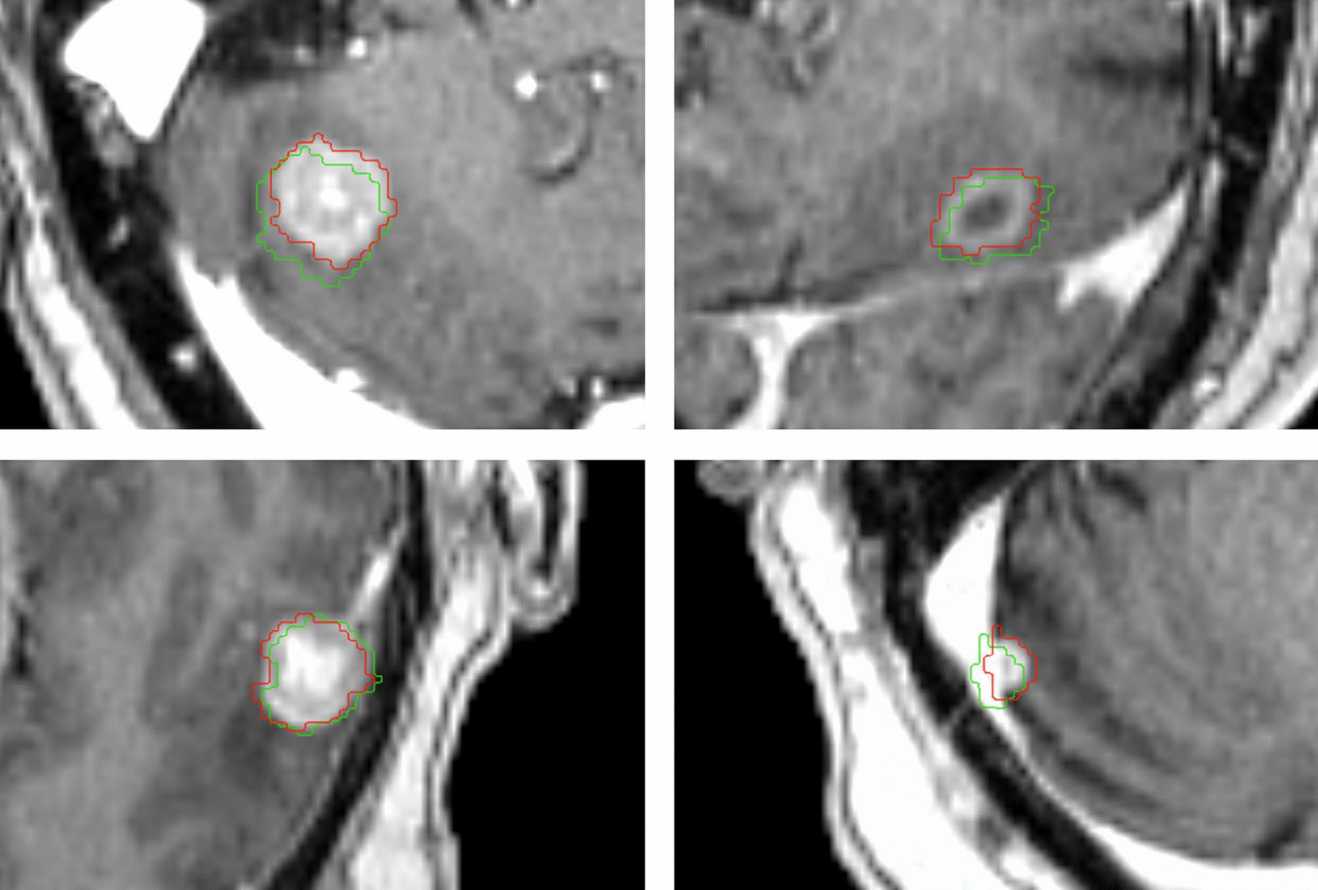
Exemplary cases from the uncorrected subgroup with considerable distortions. Uncorrected MR images are shown overlaid by uncorrected and corrected GTV contours. Red contours depict the uncorrected GTV perimeter, green contours display the corrected tumor outline after 3D distortion correction for gradient nonlinearity-induced distortions.

2D distortion-corrected MRIs had been used for treatment planning in 52.5% (220/419) of lesions, while uncorrected MRIs had been employed in the remaining 47.5% (199/419) of metastases. No instance of 3D distortion correction was identified in the present series. Figure 1 shows four exemplary brain metastases cases from the uncorrected subgroup with pronounced distortions.

**Fig. 2:**
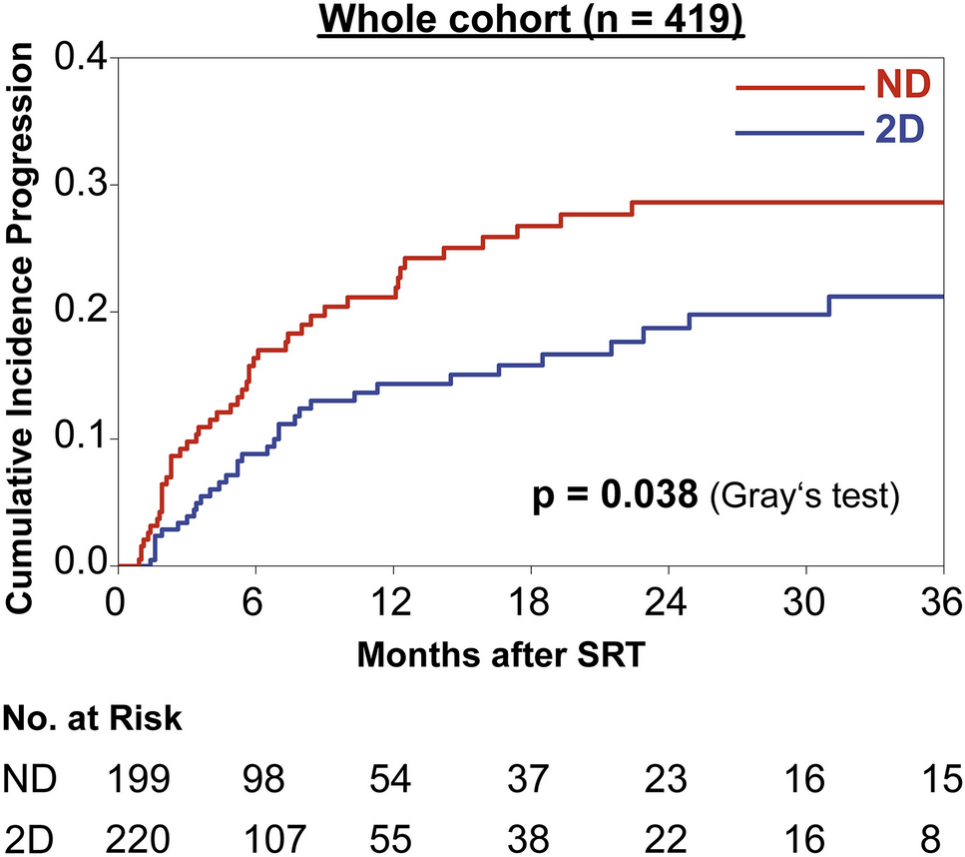
Cumulative incidence of local progression in brain metastases, in which treatment planning was based on a 2D distortion-corrected MRI (“2D”, blue, n = 220) or an uncorrected MRI dataset (“ND”, red, n = 199).

Differences in local control were assessed using the cumulative incidence method and Gray’s test to account for the competing risk of death. At 12 months post-SRT the cumulative incidence of local progression was 14.3% in the distortion-corrected group vs. 21.2% in the uncorrected subset and at 24 months the incidence of local progression was 18.7% vs. 28.6%, respectively (p = 0.038, Gray’s test, Fig. 2).

**Fig. 3:**
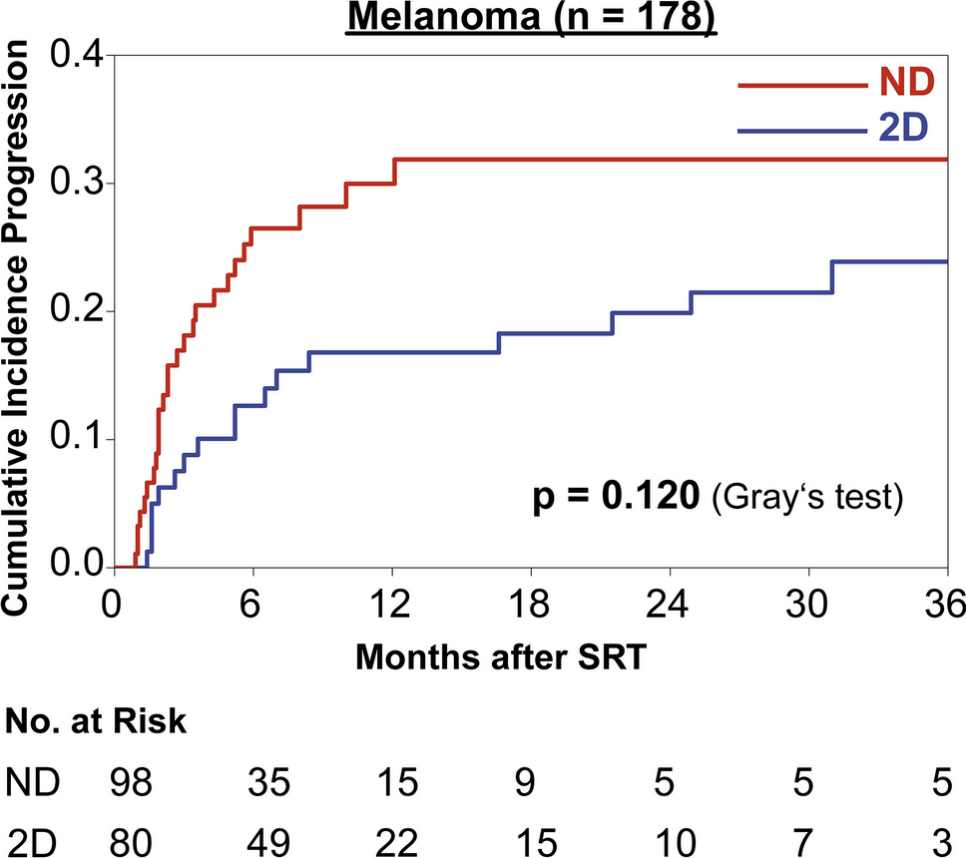
Cumulative incidence of local progression in the subset of melanoma brain metastases for treatment planning on 2D distortion corrected (“2D”, blue, n = 80) and uncorrected (“ND”, red, n = 98) MRI datasets.

To exclude confounding by primary tumor histology we performed a subgroup analysis for the largest subgroup of melanoma brain metastasis (n = 178). For melanoma brain metastases, the cumulative incidence of local progression was 16.8% vs. 30.0% at 12 months and 19.9% vs. 31.9% at 24 months for 2D-corrected and uncorrected planning MRIs, respectively (p = 0.120, Fig. 3).

When limiting the analysis to the subgroup that received stereotactic radiotherapy without adjuvant whole-brain radiotherapy (246/419), local progression was 16.2% vs. 25.5% at 12 months for corrected vs. uncorrected planning MRI (p = 0.053, Gray’s test). Conversely, in the subgroup treated with additional adjuvant whole-brain radiotherapy, local failures were reduced overall and the difference between corrected and uncorrected pretreatment MRI was less pronounced (5.1% vs. 9.6% at 12 months, p = 0.179).

Using competing risk regression, the effect of distortion correction on local control was subsequently assessed in multivariate analysis. Accounting for histology, baseline tumor volume, the time interval between MRI and treatment delivery, year the planning MRI was performed as well as for adjuvant whole-brain radiotherapy and biologically effective dose, distortion correction remained significantly associated with improved local control (HR = 0.55, p = 0.020, Table 2).

**Table 2:** Prognostic factors for local control on univariate and multivariate proportional subdistribution hazards regression analysis (Fine and Gray), n = 419.

	Univariate		Multivariate	
Parameter	HR	*p*-value	HR	*p*-value
**Distortion correction, 2D vs. ND**	**0.62**	**0.038**	**0.55**	**0.020**
**Adjuvant WBRT**	**0.42**	**0.001**	0.61	0.280
**BED**_**12-LQC**_**, Gy**	**0.97**	**0.002**	0.99	0.310
Time period of planning MRI, year	0.97	0.326	0.99	0.981
Interval between MRI and treatment, days	1.01	0.380	1.02	0.180
Baseline tumor volume, cm^3^	1.01	0.360	1.02	0.110
Radioresistant histology^R^	1.39	0.150	1.23	0.400

*HR* Hazard ratio, *WBRT* Whole-brain radiotherapy, *BED*_*12-LQC*_ Biologically effective dose for an alpha/beta ratio of 12, linear-quadratic-cubic model.

^R^Melanoma, sarcoma and renal cell carcinoma were classified as radioresistant histology.

Significant values are in bold.

**Fig. 4:**
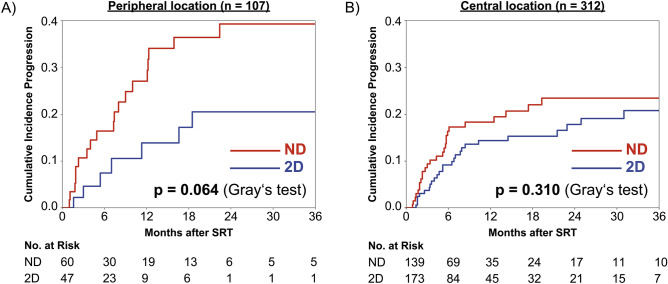
Cumulative incidence of local tumor progression according to the distortion correction status shown separately for peripheral (**A**) and centrally located lesions (**B**). increased difference between 2D distortion-corrected (“2D”, blue) and uncorrected MRI datasets (“ND”, red) in metastases with peripheral location.

The impact of distortion correction was more pronounced for peripheral compared to centrally located metastases (Fig. 4). For peripheral metastases, cumulative incidence of local progression at 12 months was 13.9% for distortion-corrected vs. 27.1% for the uncorrected planning MRI datasets (p = 0.064). Conversely, the difference at 12 months was only 14.4% vs. 18.3% for centrally located metastases (p = 0.310). In multivariate analysis including adjuvant WBRT, BED_12-LQC_, imaging-treatment time interval, tumor volume, year of the planning MRI and radioresistant histology as additional covariates, distortion correction status was a significant prognostic factor for peripheral (HR 0.34, p = 0.018) but not for centrally located metastases (HR 0.73, p = 0.324).

Most MRI datasets in the present cohort had been obtained at 1.5 T systems (90.5%), whereas only 9.5% of datasets were acquired at 3.0 T. All MR datasets acquired at 3.0 T had 2D distortion correction activated (Table 1). However, 3.0 T field strength was not a significant prognostic factor in either univariate (HR 1.09, p = 0.800) or multivariate analysis (HR 1.48, p = 0.348). Distortion correction status remained a significant prognostic factor, when field strength was added to the multivariate regression model (HR 0.51, p = 0.014). In an additional sensitivity analysis, BED was calculated according to the linear-quadratic (BED_20-LQ_) instead of the linear-quadratic-cubic model (BED_12-LQC_). For the LQ model, an α/β ratio of 20 was assumed for SRT, based on the work of Redmond et al.^45^, while an α/β ratio of 10 was applied for WBRT. The BED_20-LQ_ provided results comparable to those of the BED_12-LQC_ and was a significant prognosticator in univariate (HR 0.97, p = 0.002), but not in multivariate analysis (HR 0.99, p = 0.352). Notably, distortion correction status remained a significant prognostic factor (HR 0.55, p = 0.021), when BED_20-LQ_ was used in place of BED_12-LQC_ in the multivariate model.

**Fig. 5:**
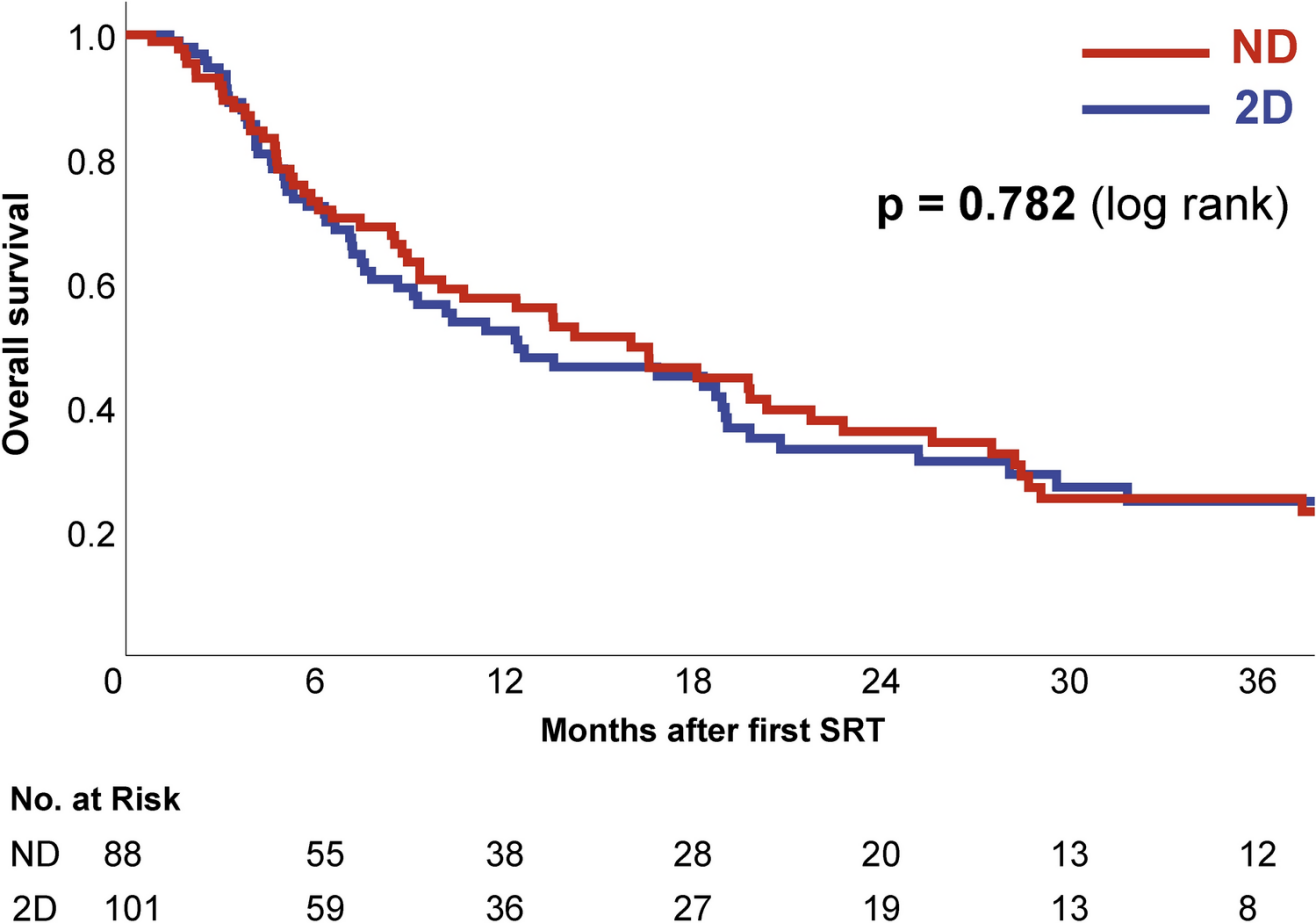
Overall survival for the distortion-corrected (“2D”, blue, n = 220) and uncorrected (“ND”, red, n = 199) subgroup.

No significant difference in overall survival was observed between the distortion-corrected and uncorrected subgroup (log rank p = 0.782). The 1-year overall survival was 52.3% for the corrected vs. 57.5% for the uncorrected subgroup while 2-year survival was calculated as 33.2% vs. 36.1% (Fig. 5).

## Discussion

While magnetic resonance imaging (MRI) for target delineation in brain tumors was introduced many decades ago^16,46–48^, the significance of pretreatment MRI quality for treatment outcomes is recently becoming recognized more widely^49–54^. In 2015, Seymour et al. were the first to demonstrate the prognostic significance of the time interval between planning MRI and SRS for subsequent local control. While they observed a local control rate of 95% at 6 months post-SRS, if the planning MRI had been performed less than 14 days before SRS, control dropped to a mere 56% if the interval was greater^13^. Since then the high relevance of up-to-date MR imaging for treatment planning has been confirmed by numerous studies^15,19,21,22^ with Salkeld et al. even reporting considerable anatomical changes within imaging intervals of less than 8 days^14^.

Gradient nonlinearity-related distortions caused by physical limits in gradient coil design generally are the most relevant type of image distortions in brain MRI^9,19,21^. As gradient nonlinearities increase with distance from the MRI isocenter, distortions intensify in centrifugal direction and may reach several millimeters at the brain surface^15,16,18,21,22,55^. While severe cases may lead to a characteristic barrel aberration^16^, more subtle distortions on the order of few millimeters are nearly impossible to identify, even when coregistering to a planning CT. Fortunately, as gradient nonlinearity-related distortions are a constant property of the gradient coil set known to the manufacturer^19,21^, they can be corrected using vendor-specific distortion correction. Vendor-specific distortion correction is typically applied as a post-processing step using an image warping technique, similar to deformable registration, which necessitates resampling and incorporates intensity correction^21,22,31^. Most modern scanners provide options for 2D and 3D distortion correction. While 2D distortion correction is limited to in-plane distortions, 3D distortion correction addresses both in-plane and through-plane distortions^26^.

A large number of important studies has been performed in various types of non-anthropomorphic phantoms to characterize distortions in MRI and to evaluate methods for correction^15,16,19,21,22,31^. However, results of these studies are frequently difficult to transfer into the clinical setting. Therefore, the actual clinical relevance of distortion correction for brain stereotactic radiotherapy has largely remained elusive to this date. This circumstance may also have contributed to an overall low awareness of MR image distortions in the general radiation oncology community. We identified one simulation study that addressed the clinical significance of MRI distortions in brain stereotactic radiotherapy. In this study, Seibert et al. compared 3D corrected with uncorrected MR images. They found a median metastasis displacement of 1.2 mm and a maximum displacement of 3.9 mm in uncorrected images. Following the results of this simulation study, geographic miss would have occurred in 8 out of 28 evaluated lesions if uncorrected MR images had been used^26^.

In a retrospective simulation study, Ohira et al. recently showed the dosimetric implications of MR distortion on brain stereotactic radiotherapy. 3D correction reduced the underdosage of the GTV significantly as a function of the distance from the isocenter. A 5% relative dose difference at the 98%-isodose was observed at 48 mm from the MR isocenter for non-corrected images compared with 70 mm for 3D-corrected images^56^.

While expert consensus and recent guidelines define vendor-specific 3D distortion correction as a minimum requirement for brain stereotactic radiotherapy to minimize gradient nonlinearity-induced distortions and to reduce total distortions to below 1 mm, corresponding clinical data are scarc^9,57^. To further elucidate the significance of distortion correction on clinical outcomes, we reviewed a historic cohort of brain metastases patients treated with stereotactic radiotherapy comparing real clinical results achieved with 2D distortion-corrected and uncorrected MRI datasets. Using competing risk analysis, we found local failure to be significantly and substantially reduced in the subset of metastases treated based on 2D distortion-corrected MRI. The two cohorts showed some imbalances across major prognostic factors for local control (Table 1). For instance, a statistically significant imbalance between single session and fractionated stereotactic radiotherapy was observed. However, while there were statistically significant differences in the distribution of the primary histology, the frequency of radioresistant histologies did not differ significantly between the two subsets. To further exclude confounding by the primary tumor histology, we performed a subgroup analysis for the largest subset of melanoma brain metastases (n = 178, Fig. 3). The subgroup analysis indicated a reduction of local failures for melanoma brain metastases with distortion correction (12-months 16.8% vs. 30.0%) and therefore showed the same overall trend as for the whole cohort. However, statistical significance could not be demonstrated (p = 0.120). To further account for known prognostic factors of local control in brain metastases, we performed a multivariate competing risk analysis, in which distortion correction remained significantly associated with improved local control. To adjust for potentially confounding changes in imaging or treatment delivery over the 12-year study period, the year of the planning MRI was included in the multivariate analysis. The year of the planning MRI was not found to be a significant prognostic factor in univariate or multivariate analysis (HR 0.97, p = 0.326 and HR 0.99, p = 0.981 respectively), while distortion correction remained the most important multivariate prognostic factor (HR 0.55, p = 0.020). Finally, our analysis revealed that the impact of distortion correction was significantly more pronounced for peripheral brain metastases compared to those located centrally (Fig. 4). Since gradient non-linearity-related distortions intensify with increasing distance from the scanner isocenter, this observation further supports the causal relationship between enhanced geometric accuracy achieved through distortion correction and improved clinical outcomes. This study therefore is the first to provide clinical evidence that distortion-correction does improve local control in brain stereotactic radiotherapy. This adds to the growing body of literature indicating that optimal pretreatment MR imaging substantially influences actual clinical outcomes in radiation oncology.

However, no significant impact of 2D correction on overall survival was observed in the present cohort (12 months OS, 52.3% vs 57.5%, p = 0.782). This finding aligns with existing literature indicating that enhanced local control of brain metastases does not necessarily translate into improved overall survival for an unselected population^58^. Furthermore, there are methodological limitations regarding the assessment of overall survival in this cohort. For instance, important prognostic parameters such as systemic tumor burden and extracerebral tumor control were not captured. Additionally, the long observation period of over 12 years hinders interpretation of survival outcomes and the statistical power was not sufficient to detect significant differences in overall survival.

Consistent with previous studies, we found that increasing time intervals between MRI and stereotactic radiotherapy were associated with reduced local control (HR 1.02 per day in multivariate analysis). However, this parameter did not reach significance in the present analysis (p = 0.180 in multivariate analysis), which can be explained by the fact that the median interval between stereotactic radiotherapy and planning MRI was only 9 days. The number of lesions with very high time interval consecutively might have been too low to achieve statistical significance. Some patients received WBRT prior to SRT which could have allowed tumor growth or swelling, which in terms could have affected SRT precision. To account for this, we included WBRT in the multivariate analysis, where the effect of distortion correction remained significant. Mechanistically, distortion correction is unlikely to affect the efficacy of adjuvant WBRT. However, irrespective of the delivery of adjuvant WBRT, the use of uncorrected MRI datasets for treatment planning compromises the accuracy of SRT. Supporting this assumption, the application of 2D distortion correction was also associated with improved local control within the WBRT subgroup in this series (5.1% vs. 9.6% at 12 months), though the difference did not reach statistical significance (p = 0.179).

It has to be noted that distortion correction could have achieved improved local control not only by a more accurate depiction of real tumor boundaries that avoided marginal miss as in the study by Seibert et al., but also by improvements in MRI-to-CT coregistration, that may have been impaired by distortions near the brain and skull surface as well as accompanying shifts in signal intensity in uncorrected images. We are currently comprehensively investigating these mechanistic questions that were out of the scope of the present work.

While we were only able to evaluate the effects of 2D distortion correction in this historic series, it has to be stressed that vendor-specific 3D distortion correction is currently considered a minimum requirement for radiotherapy treatment planning^9,57^. Enabling vendor-specific 3D distortion correction at the MR scanner unfortunately is not an all-in-one solution to comprehensively address all distortions in MR images, however. Even after vendor-specific 3D correction, residual gradient nonlinearity-related distortions may remain that could require additional correction. Current expert consensus therefore recommends to characterize these residual distortions via phantom measurements and to apply additional corrections if necessary^9^.

Moreover, additional types of MRI distortions that are not addressed by distortion correction are relevant for brain stereotactic radiotherapy. Inhomogeneities in the main magnetic field, which arise because of residual magnet imperfections and because of magnetic field perturbations individually induced by the patient anatomy itself, also cause distortions^9,59,60^. Instead of applying distortion correction as a post-processing step these distortions can be addressed by patient-specific active shimming and by setting an RT-optimized pixel-bandwidth during sequence acquisition^9,25^. Beyond distortion correction, numerous quality requirements for MRI simulation in cranial stereotactic radiotherapy have been outlined in recent guidelines^57^. These include selecting optimal sequence protocols, defining radiotherapy-optimized sequence protocol parameters, minimizing imaging-to-treatment intervals, performing MRI in the radiotherapy treatment position, and conducting regular quality assurance, among other measures^57^. Dedicated MRI simulation programs for radiotherapy treatment planning have recently emerged as an interesting model to comprehensively address these requirements and have been established at multiple centers^59,61^.

## Limitations

Due to the retrospective nature of this study, confounding effects cannot be excluded. Imbalances between the 2D corrected and uncorrected subset could have influenced results. Influences of additional confounders such as the long investigational interval of 12 years, outdated patient cohort, heterogeneity of used MR scanner models, differences in radiotherapy techniques and systemic treatments could have impacted the results. In this retrospective analysis, frequency and modality (1.5 T, 3 T) of the acquired follow-up MRs was not standardized and could have affected the outcomes. Also hidden confounders are possible in retrospective series. However, no other study design would have been ethically acceptable.

## Conclusion

This is the first study to evaluate the impact of MRI distortion correction on local control in stereotactic radiotherapy for brain metastases. In this historic series, we found significantly higher local control when using 2D corrected vs. uncorrected MRI studies for treatment planning. These results stress the importance of assuring that MR images used for radiotherapy treatment planning are properly distortion-corrected. Recent expert-based guidelines should be followed when using MR imaging for radiotherapy target delineation^57,61^. Optimizing MRI for radiotherapy treatment planning might be an important area of research to further improve patient outcomes in radiation oncology.

## Data Availability

The analyzed data are available from the corresponding author upon reasonable request.

## References

[1] Tabouret, E. et al. Recent trends in epidemiology of brain metastases: an overview. *Anticancer Res.* **32**(11), 4655–4662 (2012). 23155227

[2] Soffietti, R. et al. Diagnosis and treatment of brain metastases from solid tumors: guidelines from the European association of neuro-oncology (EANO). *Neuro Oncol.* **19**(2), 162–174 (2017). 28391295 10.1093/neuonc/now241 PMC5620494

[3] Gaudy-Marqueste, C. et al. Survival of melanoma patients treated with targeted therapy and immunotherapy after systematic upfront control of brain metastases by radiosurgery. *Eur. J. Cancer* **84**, 44–54 (2017). 28783540 10.1016/j.ejca.2017.07.017

[4] Johung, K. L. et al. Extended survival and prognostic factors for patients with ALK-rearranged non-small-cell lung cancer and brain metastasis. *J. Clin. Oncol.* **34**(2), 123–129 (2016). 26438117 10.1200/JCO.2015.62.0138 PMC5070549

[5] Steinmann, D. et al. Quality of life in patients with limited (1–3) brain metastases undergoing stereotactic or whole brain radiotherapy: A prospective study of the DEGRO QoL working group. *Strahlenther. Onkol.* **196**(1), 48–57 (2020). 31418046 10.1007/s00066-019-01506-w

[6] Brown, P. D. et al. Effect of radiosurgery alone vs radiosurgery with whole brain radiation therapy on cognitive function in patients with 1 to 3 brain metastases: A randomized clinical trial. *JAMA* **316**(4), 401–409 (2016). 27458945 10.1001/jama.2016.9839 PMC5313044

[7] Aoyama, H. et al. Stereotactic radiosurgery plus whole-brain radiation therapy vs stereotactic radiosurgery alone for treatment of brain metastases: a randomized controlled trial. *JAMA* **295**(21), 2483–2491 (2006). 16757720 10.1001/jama.295.21.2483

[8] Kocher, M. et al. Adjuvant whole-brain radiotherapy versus observation after radiosurgery or surgical resection of one to three cerebral metastases: results of the EORTC 22952–26001 study. *J. Clin. Oncol.* **29**(2), 134–141 (2011). 21041710 10.1200/JCO.2010.30.1655 PMC3058272

[9] Paulson, E. S. et al. Consensus opinion on MRI simulation for external beam radiation treatment planning. *Radiother. Oncol.* **121**(2), 187–192 (2016). 27838146 10.1016/j.radonc.2016.09.018

[10] Schmitt, D. et al. Technological quality requirements for stereotactic radiotherapy: Expert review group consensus from the DGMP working group for physics and technology in stereotactic radiotherapy. *Strahlenther. Onkol.* **196**(5), 421–443 (2020). 32211939 10.1007/s00066-020-01583-2 PMC7182540

[11] Hessen, E. et al. Predicting and implications of target volume changes of brain metastases during fractionated stereotactic radiosurgery. *Radiother. Oncol.*10.1016/j.radonc.2019.07.011 (2019). 31431379 10.1016/j.radonc.2019.07.011

[12] Hessen, E. D. et al. Significant tumor shift in patients treated with stereotactic radiosurgery for brain metastasis. *Clin. Transl. Radiat. Oncol.* **2**, 23–28 (2017). 29657996 10.1016/j.ctro.2016.12.007 PMC5893526

[13] Seymour, Z. A. et al. Interval from imaging to treatment delivery in the radiation surgery age: How long is too long?. *Int. J. Radiat. Oncol. Biol. Phys.* **93**(1), 126–132 (2015). 26279030 10.1016/j.ijrobp.2015.05.001

[14] Salkeld, A. L. et al. Changes in brain metastasis during radiosurgical planning. *Int. J. Radiat. Oncol. Biol. Phys.* **102**(4), 727–733 (2018). 29953911 10.1016/j.ijrobp.2018.06.021

[15] Baldwin, L. N. et al. Characterization, prediction, and correction of geometric distortion in 3 T MR images. *Med. Phys.* **34**(2), 388–399 (2007). 17388155 10.1118/1.2402331

[16] Sumanaweera, T. S. et al. Characterization of spatial distortion in magnetic resonance imaging and its implications for stereotactic surgery. *Neurosurgery* **35**(4), 696–703 (1994). 7808613 10.1227/00006123-199410000-00016

[17] Reinsberg, S. A. et al. A complete distortion correction for MR images: II. Rectification of static-field inhomogeneities by similarity-based profile mapping. *Phys. Med. Biol.* **50**(11), 2651–61 (2005). 15901960 10.1088/0031-9155/50/11/014

[18] Baldwin, L. N., Wachowicz, K. & Fallone, B. G. A two-step scheme for distortion rectification of magnetic resonance images. *Med. Phys.* **36**(9), 3917–3926 (2009). 19810464 10.1118/1.3180107

[19] Doran, S. J. et al. A complete distortion correction for MR images: I. Gradient warp correction. *Phys. Med. Biol.* **50**(7), 1343–1361 (2005). 15798328 10.1088/0031-9155/50/7/001

[20] Fransson, A., Andreo, P. & Potter, R. Aspects of MR image distortions in radiotherapy treatment planning. *Strahlenther. Onkol.* **177**(2), 59–73 (2001). 11233837 10.1007/pl00002385

[21] Karger, C. P. et al. Accuracy of device-specific 2D and 3D image distortion correction algorithms for magnetic resonance imaging of the head provided by a manufacturer. *Phys. Med. Biol.* **51**(12), N253–N261 (2006). 16757858 10.1088/0031-9155/51/12/N04

[22] Jovicich, J. et al. Reliability in multi-site structural MRI studies: effects of gradient non-linearity correction on phantom and human data. *Neuroimage* **30**(2), 436–443 (2006). 16300968 10.1016/j.neuroimage.2005.09.046

[23] Torfeh, T. et al. Characterization of 3D geometric distortion of magnetic resonance imaging scanners commissioned for radiation therapy planning. *Magn. Reson. Imaging* **34**(5), 645–653 (2016). 26795695 10.1016/j.mri.2016.01.001

[24] Stanescu, T. et al. A study on the magnetic resonance imaging (MRI)-based radiation treatment planning of intracranial lesions. *Phys. Med. Biol.* **53**(13), 3579–3593 (2008). 18560047 10.1088/0031-9155/53/13/013

[25] Putz, F. et al. Magnetic resonance imaging for brain stereotactic radiotherapy: A review of requirements and pitfalls. *Strahlenther. Onkol.* **196**(5), 444–456 (2020). 32206842 10.1007/s00066-020-01604-0 PMC7182639

[26] Seibert, T. M. et al. Distortion inherent to magnetic resonance imaging can lead to geometric miss in radiosurgery planning. *Pract. Radiat. Oncol.* **6**(6), e319–e328 (2016). 27523440 10.1016/j.prro.2016.05.008 PMC5099096

[27] Koca, S. et al. Time course of pain response and toxicity after whole-nerve-encompassing LINAC-based stereotactic radiosurgery for trigeminal neuralgia-a prospective observational study. *Strahlenther. Onkol.* **195**(8), 745–755 (2019). 30877350 10.1007/s00066-019-01450-9

[28] Putz, F. et al. Stereotactic radiotherapy of vestibular schwannoma : Hearing preservation, vestibular function, and local control following primary and salvage radiotherapy. *Strahlenther. Onkol.* **193**(3), 200–212 (2017). 27928625 10.1007/s00066-016-1086-5

[29] Wiggenraad, R. et al. Dose-effect relation in stereotactic radiotherapy for brain metastases. A systematic review. *Radiother. Oncol.* **98**(3), 292–297 (2011). 21316787 10.1016/j.radonc.2011.01.011

[30] Joiner, Quantifying cell kill and survival, in Basic clinical radiobiology. In *Basic Clinical Radiobiology* (ed. Joiner, M.) (CRC Press, 2009).

[31] Janke, A. et al. Use of spherical harmonic deconvolution methods to compensate for nonlinear gradient effects on MRI images. *Magn. Reson. Med.* **52**(1), 115–122 (2004). 15236374 10.1002/mrm.20122

[32] Putz, F. et al. FSRT vs SRS in brain metastases – Differences in local control and radiation necrosis – a volumetric study. *Front. Oncol.*10.3389/fonc.2020.559193 (2020). 33102223 10.3389/fonc.2020.559193 PMC7554610

[33] Lin, N. U. et al. Response assessment criteria for brain metastases: proposal from the RANO group. *Lancet Oncol.* **16**(6), e270–e278 (2015). 26065612 10.1016/S1470-2045(15)70057-4

[34] Scrucca, L., Santucci, A. & Aversa, F. Regression modeling of competing risk using R: an in depth guide for clinicians. *Bone Marrow Transpl.* **45**(9), 1388–1395 (2010). 10.1038/bmt.2009.359 20062101

[35] Gray, R. J. A class of K-sample tests for comparing the cumulative incidence of a competing risk. *Ann. Stat.*10.1214/aos/1176350951 (1988).

[36] Fine, J. P. & Gray, R. J. A proportional hazards model for the subdistribution of a competing risk. *J. Am. Stat. Assoc.* **94**(446), 496–509 (1999).

[37] Nardone, V. et al. Role of perilesional edema and tumor volume in the prognosis of non-small cell lung cancer (NSCLC) undergoing radiosurgery (SRS) for brain metastases. *Strahlenther. Onkol.* **195**(8), 734–744 (2019). 31123785 10.1007/s00066-019-01475-0

[38] Brown, P. D. et al. Stereotactic radiosurgery for patients with “radioresistant” brain metastases. *Neurosurgery* **51**(3), 656–65 (2002). 12188943

[39] Kaplan, E. L. & Meier, P. Nonparametric estimation from incomplete observations. *J. Am. Stat. Assoc.* **53**(282), 457–481 (1958).

[40] Mantel, N. Evaluation of survival data and two new rank order statistics arising in its consideration. *Cancer Chemother. Rep.* **50**(3), 163–170 (1966). 5910392

[41] Peto, R. & Peto, J. Asymptotically efficient rank invariant test procedures. *J. Royal Stat. Soc. Ser. A (Gen.)* **135**(2), 185–207 (1972).

[42] Isensee, F. et al. Automated brain extraction of multisequence MRI using artificial neural networks. *Hum. Brain Mapp.* **40**(17), 4952–4964 (2019). 31403237 10.1002/hbm.24750 PMC6865732

[43] Fedorov, A. et al. 3D Slicer as an image computing platform for the quantitative imaging network. *Magn. Reson. Imaging* **30**(9), 1323–1341 (2012). 22770690 10.1016/j.mri.2012.05.001 PMC3466397

[44] Schroeder, W., Martin, K. & Lorensen, B. *The Visualization Toolkit* 4th edn. (Kitware, 2006).

[45] Redmond, K. J. et al. Tumor control probability of radiosurgery and fractionated stereotactic radiosurgery for brain metastases. *Int. J. Radiat. Oncol. Biol. Phys.* **110**(1), 53–67 (2021). 33390244 10.1016/j.ijrobp.2020.10.034

[46] Potter, R. et al. Sagittal and coronal planes from MRI for treatment planning in tumors of brain, head and neck: MRI assisted simulation. *Radiother. Oncol.* **23**(2), 127–130 (1992). 1546188 10.1016/0167-8140(92)90344-t

[47] Khoo, V. S. et al. Magnetic resonance imaging (MRI): considerations and applications in radiotherapy treatment planning. *Radiother. Oncol.* **42**(1), 1–15 (1997). 9132820 10.1016/s0167-8140(96)01866-x

[48] Kondziolka, D. et al. A comparison between magnetic resonance imaging and computed tomography for stereotactic coordinate determination. *Neurosurgery* **30**(3), 402–6 (1992). 1620305 10.1227/00006123-199203000-00015

[49] Welzel, T. et al. Stereotactic radiotherapy of brain metastases: clinical impact of three-dimensional SPACE imaging for 3T-MRI-based treatment planning. *Strahlenther. Onkol.*10.1007/s00066-022-01996-1 (2022). 35976408 10.1007/s00066-022-01996-1 PMC9515140

[50] Mekiš, V. et al. Comparison of treatment position with mask immobilization and standard diagnostic setup in intracranial MRI radiotherapy simulation. *Strahlenther. Onkol.* **197**(7), 614–621 (2021). 33881558 10.1007/s00066-021-01776-3

[51] Mengling, V. et al. Implementation of a dedicated 1.5 T MR scanner for radiotherapy treatment planning featuring a novel high-channel coil setup for brain imaging in treatment position. *Strahlenther. Onkol.* **197**(3), 246–256 (2021). 33103231 10.1007/s00066-020-01703-y PMC7892740

[52] Glide-Hurst, C. K. et al. Task group 284 report: magnetic resonance imaging simulation in radiotherapy: considerations for clinical implementation, optimization, and quality assurance. *Med. Phys.* **48**(7), e636–e670 (2021). 33386620 10.1002/mp.14695 PMC8761371

[53] Welzel, T. et al. Stereotactic radiotherapy of brain metastases: clinical impact of three-dimensional SPACE imaging for 3T-MRI-based treatment planning. *Strahlenther. Onkol.* **198**(10), 926–933 (2022). 35976408 10.1007/s00066-022-01996-1 PMC9515140

[54] Masitho, S. et al. Synthetic CTs for MRI-only brain RT treatment: integration of immobilization systems. *Strahlenther. Onkol.* **199**(8), 739–748 (2023). 37285037 10.1007/s00066-023-02090-w PMC10361877

[55] Paulson, E. S. et al. Comprehensive MRI simulation methodology using a dedicated MRI scanner in radiation oncology for external beam radiation treatment planning. *Med. Phys.* **42**(1), 28–39 (2015). 25563245 10.1118/1.4896096

[56] Ohira, S. et al. Impact of magnetic resonance imaging-related geometric distortion of dose distribution in fractionated stereotactic radiotherapy in patients with brain metastases. *Strahlenther. Onkol.*10.1007/s00066-023-02120-7 (2023). 37591978 10.1007/s00066-023-02120-7

[57] Putz, F. et al. Quality requirements for MRI simulation in cranial stereotactic radiotherapy: a guideline from the German Taskforce “Imaging in Stereotactic Radiotherapy”. *Strahlenther. Onkol.* **200**(1), 1–18 (2024). 38163834 10.1007/s00066-023-02183-6 PMC10784363

[58] Andrews, D. W. et al. Whole brain radiation therapy with or without stereotactic radiosurgery boost for patients with one to three brain metastases: phase III results of the RTOG 9508 randomised trial. *Lancet* **363**(9422), 1665–1672 (2004). 15158627 10.1016/S0140-6736(04)16250-8

[59] Mengling, V. et al. Evaluation of the influence of susceptibility-induced magnetic field distortions on the precision of contouring intracranial organs at risk for stereotactic radiosurgery. *Phys. Imaging Radiat. Oncol.* **15**, 91–97 (2020). 33458332 10.1016/j.phro.2020.08.001 PMC7807629

[60] Wang, H., Balter, J. & Cao, Y. Patient-induced susceptibility effect on geometric distortion of clinical brain MRI for radiation treatment planning on a 3T scanner. *Phys. Med. Biol.* **58**(3), 465–477 (2013). 23302471 10.1088/0031-9155/58/3/465

[61] Moore-Palhares, D. et al. Clinical implementation of magnetic resonance imaging simulation for radiation oncology planning: 5 year experience. *Radiat. Oncol.* **18**(1), 27 (2023). 36750891 10.1186/s13014-023-02209-4 PMC9903411

